# Dexmedetomidine Improves Learning Functions in Male Rats Modeling Cognitive Impairment by Modulating the BDNF/TrkB/CREB Signaling Pathway

**DOI:** 10.3390/life14121672

**Published:** 2024-12-17

**Authors:** Sinan Saral, Tolga Mercantepe, Atilla Topçu, Ali Koray Kaya, Aykut Öztürk

**Affiliations:** 1 Department of Physiology, Faculty of Medicine, Recep Tayyip Erdogan University, 53100 Rize, Türkiye; 2 Department of Histology and Embryology, Faculty of Medicine, Recep Tayyip Erdogan University, 53100 Rize, Türkiye; tolga.mercantepe@erdogan.edu.tr; 3 Department of Medical Pharmacology, Faculty of Medicine, Recep Tayyip Erdogan University, 53100 Rize, Türkiye; 4 Department of Physiology, Faculty of Medicine, Kütahya Health Sciences University, 43020 Kütahya, Türkiye; alikoray.kaya@ksbu.edu.tr; 5 Department of Pharmacology, Ministry of Health, Derince Education and Research Hospital, 41100 Kocaeli, Türkiye; draykutoz@gmail.com

**Keywords:** scopolamine, cognitive impairment, dexmedetomidine, TrkB, BDNF, CREB

## Abstract

Dexmedetomidine (DEX) is a selective alpha-2 adrenergic receptor agonist with sedative and anxiolytic properties. Increasing evidence reports that DEX has a neuroprotective effect. In this study, we investigated the potential effects of DEX on learning and memory functions in rats with experimental cognitive impairment. In the study, 21 adult male rats were used. The rats were divided into three groups, namely control, Scopolamine (SCOP) and SCOP + DEX. Cognitive impairment was induced with 1 mg/kg SCOP daily for 21 days. DEX was administered at a dose of 10 µg/kg between days 14 and 21 of the experiment. Following the injections, a spatial memory test was performed with a Morris Water Maze (MWM). At the end of the experiment, the hippocampus was dissected. The brain-derived neurotrophic factor (BDNF), acetylcholine (ACh) and acetylcholinesterase (AChE) levels were determined by ELISA. The tropomyosin receptor kinase B (TrkB) and Cyclic AMP-Response Element-Binding Protein (CREB) levels were measured by immunohistochemistry. DEX treatment improved the learning performance of rats compared to SCOP for 5 days. However, it did not significantly change memory performance. DEX increased the BDNF and ACh levels in the hippocampus while decreasing the AChE levels. Similarly, DEX treatment significantly increased CREB phosphorylation. No significant difference was observed between the TrkB receptor levels of the groups. This study demonstrated that the role of DEX in reducing SCOP-induced cognitive impairment is partially mediated by the increase in BDNF/TrkB/CREB signaling pathway activity.

## 1. Introduction

The most common type of disease characterized by dementia is Alzheimer’s disease (AD). AD is reported to affect more than 35 million people worldwide [1]. In AD, cognitive impairment, mental dysfunction, personality and behavioral changes are mainly observed [2]. The disease is associated with progressive cognitive decline and the accumulation of amyloid-beta (Aβ) and tau proteins. Moreover, at the center of the clinical manifestations of the disease is the loss of synaptic plasticity and the degeneration of synapses, which are closely associated with cognitive decline [3]. BDNF, one of the most important neurotrophins of the central nervous system, plays an active role in synaptic plasticity. BDNF and its receptor tropomyosin receptor kinase B (TrkB) have been implicated in the control of neurotransmitter release, ion channel activity, axonal transmission and neuronal excitability [4]. Neuronal apoptosis, increased tau phosphorylation, and neuroinflammation have been associated with decreased BDNF levels [5]. Increasing evidence suggests that Aβ reduces BDNF by downregulating phosphorylated cyclic adenosine monophosphate (cAMP) response element-binding protein (CREB) [6]. 

The degeneration of cholinergic neurons is critical in the pathophysiology of AD. Acetylcholine (ACh), a neurotransmitter secreted by cholinergic neurons, plays a key role in signalling related to memory and learning ability [1]. Cholinergic atrophy and cognitive decline are among the most important indicators in age-related neurodegenerative diseases such as AD [7]. It has been reported that abnormalities of the cholinergic system can lead to neurotoxicity, neuronal inflammation and cell damage through amyloid precursor protein (APP) metabolism and tau phosphorylation [8]. Nicotinic and muscarinic ACh receptors are targeted in the treatment of cognitive deficits in some neurodegenerative disorders including AD [9]. However, due to the complex and uncertain pathogenesis, there are no completely effective methods able to prevent and treat AD. Therefore, there is an increasing need for research on compounds with the potential to modulate the cholinergic system.

Scopolamine (SCOP) is a nonselective muscarinic cholinergic receptor antagonist that can cause cognitive deficits, including in short- and long-term memory, in humans and rodents [10,11]. A recent study reported that scopolamine is associated with discriminative and spatial memory deficits [12]. SCOP is widely used in experimental neurocognitive studies because scopolamine administration reflects both the behavioral and molecular features of Alzheimer’s disease and other neurocognitive disorders. At the molecular level, SCOP is characterized by imbalanced cholinergic transmission in the hippocampus and prefrontal cortex [13]. Furthermore, SCOP was found to increase AChE activity in the cortex and hippocampus. A recent study reported that SCOP decreased BDNF expression and suppressed TrkB, ERK and CREB phosphorylation in the hippocampus [14]. These results confirm that SCOP is a pharmacological agent that can be used to induce learning and memory impairment in rodents [15]. 

Dexmedetomidine (DEX) is an α2-adrenergic receptor agonist and has mainly sedative, analgesic and anxiolytic effects [16]. Increasing evidence suggests that DEX may be a potential neuroprotective agent [17,18]. Previous studies have revealed the importance of the activation of the BDNF signaling pathway in the mechanisms mediating the neuroprotective effects of DEX [19,20]. A recent study reported that DEX alleviated cognitive impairment in hypoxic–ischemic neonatal rats by promoting hippocampal neurogenesis via the BDNF/TrkB/CREB signaling pathway [21]. Additionally, there is evidence to suggest that DEX may modulate the cholinergic system [22]. These results point to the potential role of DEX in preventing AD-related cognitive dysfunction. 

The aim of this study was to investigate the potential effects of DEX on the brain BDNF/TrkB/CREB signaling pathway and cholinergic system in rats with SCOP-induced memory deficits. For this purpose, we investigated the effects of DEX on impaired brain neurochemistry and cognitive functions by neurobehavioral, biochemical and immunohistochemical methods. Our study provides new evidence that DEX treatment alleviates SCOP-induced cognitive impairment in adult rats.

## 2. Materials and Methods

### 2.1. Experimental Animals

Twenty-one male Sprague Dawley rats, 12–14 weeks old and weighing 300 ± 20 g, were used in the study. All animals were housed in accordance with the principles outlined in the Guide for the Care and Use of Laboratory Animals. In addition, local ethics committee approval was obtained (approval number 2023/25). Throughout the study, rats were housed in standard plastic cages on a sawdust floor under standard room temperature (23 °C), humidity (55–65%) and light (12 h dark/12 h light cycle) conditions. Seven rats were kept in each cage (n = 7). Except for in behavioral tests, no food restriction was applied to the rats.

### 2.2. Chemicals

Scopolamine hydrobromide was purchased from Sigma-Aldrich (Sigma–Aldrich Corp., Darmstadt, Germany) and DEX (Precedex 200 µg/2 mL) was purchased from Meditera İthalat ve İhracat Anonim Şirketi, Istanbul, Turkey. Ketamine hydrochloride (Ketalar, 90 mg/10 mL, Pfizer İlaçları Ltd. Şti., Istanbul, Turkey) and the sedative xylazine hydrochloride (Rompun, 2% 25 mL, Bayer Türk Kimya Sanayii A.Ş., Istanbul, Turkey) were used for anesthesia.

### 2.3. Experimental Protocol

The rats were randomly allocated into 3 groups with seven animals (n = 7/group). The control group received a single dose of intraperitoneal (i.p) saline solution every day for 3 weeks. SCOP was administered at a dose of 1 mg/kg i.p once daily for 3 weeks to induce cognitive impairment [22]. The SCOP + DEX group was injected with 10 µg/kg i.p. 30 min after 1 mg/kg/day SCOP administration between days 14 and 21 of the experiment. All injections were completed on day 21. Behavioral tests were performed for 6 days following the end of the injections. At the end of the experiment, all rats were sacrificed with terminal anesthesia. Brain tissues were stored at −80 °C for biochemical analyses.

### 2.4. Cognitive Test

#### Morris Water Maze (MWM)

The MWM test was performed based on our previous study [23]. In the experiment, a swimming tank with a diameter of 150 cm and a height of 50 cm was divided into 4 equal parts in a behavioral software program (ANY maze 7.40 version, Dublin/Ireland). An escape platform with a diameter of 15 cm was placed at the midpoint of the north quadrant of the tank. The temperature of the water was set to 23 ± 2 °C. During the pre-training phase (day 0), the rats were released into the water and were expected to reach the escape platform 1 cm above the water within 60 s. When the platform could not be found, the rats were guided to reach the platform. To facilitate the rats’ learning, cues containing geometric shapes (triangles, etc.) were placed around the platform. After finding the platform, the rats were allowed to stay there for 30 s. During the training phase, the water in the tank was changed daily and made opaque with milk powder. The height of the platform was then lowered by 2 cm to facilitate invisibility. Then, the rats were made to swim for 60 s with 3 repetitions per day for 5 days (acquisition trials). The time spent by the rats finding the hidden platform for 5 days was scored as the escape latency. On day 6 (probe trial), the platform was removed from the water and the time the rats spent in the target quadrant in a single swimming session was recorded. Test recordings were made with an overhead video camera system in a sound-isolated environment. In the analyses, the escape latency (s), time spent in the target quadrant (s), number of entries into the target quadrant and latency to first entry into the target quadrant (s) were evaluated to assess the rats’ learning and memory performance.

### 2.5. Biochemical Analysis Procedures

#### 2.5.1. Tissue Sample Collection and Homogenisation

The rats’ hippocampus tissue was carefully separated from their brain tissue. Phosphate buffer (PBS, pH: 7.4) (Sigma-Aldrich Corp., Darmstadt, Germany) was then added to the tissues at twice its weight. Homogenization was then performed for 5 min at 30 Hertz using a Tissue Lyser II device (Qiagen, Hilden, Germany). Following these procedures, the tissues were centrifuged at 3000× *g* for fifteen minutes and supernatants were prepared [1].

#### 2.5.2. BDNF, ACh and AChE Analysis

The BDNF, ACh and AChE levels in the hippocampal tissue were analyzed using rat-specific ELISA kits (Catalogue Nos. E-EL-R1235, E-EL-0081 and E-EL-R0355, respectively, Elabscience, Houston, TX, USA). The sensitivity and detection ranges were 18.75 pg/mL, 9.38 pg/mL and 0.47 ng/mL, respectively. The concentrations of the parameters of interest were measured in triplicate.

### 2.6. Histopathological Analysis

The hippocampus tissue samples extracted from anesthetized rats in a cold chain were kept in 10% phosphate-buffered neutral formalin (pH: 7.4, Merck KGAa, Darmstadt, Germany) for 24–36 h. Afterwards, the tissue samples were fixed and dehydrated, cleared and kept in paraffin using a tissue-tracking device (Citadel 2000, ThermoScientetic, Dreieich, Germany). In the next step, the hippocampus tissue samples were embedded in tissue-embedding cassettes (Isloab GmbH, Eschau, Germany) with paraffin using a tissue-embedding device (Leica EG1150 H, Leica Biosystems, Nussloch, Germany). Then, 4–5 µm sections were taken from the obtained tissue samples using a rotary microtome (RM2525, Leica Biosystems, Nussloch, Germany). The sections obtained were stained with a tissue-tracking device (Leica ST1050, Lecia Biosystems, Germany), Harris hematoxylin (Merck KGAa, Darmstadt, Germany) and Eosin G (Merck KGAa, Darmstadt, Germany) (Leica ST1050, Lecia Biosystems, Nussloch, Germany). The sections were coverslipped with Entellan (Merck KGAa, Darmstadt, Germany) and photographed under a light microscope (BX51, Olympus Corp., Tokyo, Japan) with a digital camera attachment (DP71, Olympus DP71, Olympus Corp., Tokyo, Japan).

### 2.7. Immunohistochemistry (IHC) Analysis

To identify damaged neurons, primary antibody anti-TrkB (ab181580, Abcam, Cambridge, UK) and CREB (ab32096, Abcam, Cambridge, UK) kits were used in an automated IHC staining device. For the IHC method, hippocampus tissue sections were cut using a rotary microtome (RM2525, Leica Biosystems, Nussloch, Germany) with 1–3 µm sections. The hippocampus tissue sections were incubated with primary and secondary antibodies using an IHC stainer device (Bond Max, Leica Biosystems, Nussloch, Germany), in accordance with the manufacturer’s instructions, for 60 min. Then, Leica Ultraview (Leica Biosystems, Nussloch, Germany) incubation and Harris hematoxylin staining were performed, respectively. Then, the sections were covered with entellan.

### 2.8. Semi-Quantitative Analysis

H+E-stained sections were scored as described in our previous studies (Table 1) [23]. The hippocampus tissue sections were evaluated by a double-blind histopathologist, with each sample under a light microscope with 40 randomly selected fields at ×40 objective magnification. Neurons showing immuno-positivity for TrkB and CREB primary antibodies by immunohistochemical methods were scored by a double-blind histopathologist in 40 randomly selected fields under a light microscope at ×40 objective magnification (Table 2).

### 2.9. Statistical Analysis

The escape latency measured in MWM was assessed by a two-way ANOVA followed by a post hoc Bonferroni test. For the other behavioral parameters of the study and ELISA analyses, one-way ANOVA followed by a post hoc Tukey test was used. A Shapiro–Wilk test and Levene’s test were performed, a Q-Q graph was created, and the Skewness–Kurtosis values were considered for the data obtained from the immunohistochemical and histopathological analyses using SPSS 20 (IBM, Armonk, NY, USA) statistical software. The parametric data were calculated as standard deviation and analyzed using one-way ANOVA and the Bonferroni test. Nonparametric data were calculated as median in the 25% and 75% interquartile range and analyzed using the Mann–Whitney U test with Bonferroni correction. Graphical plots were performed using GraphPad Prism (Version 6.0, San Diego, CA, USA). *p* < 0.05 was considered statistically significant.

## 3. Results

### 3.1. Morris Water Maze (MWM)

**Figure 1:**
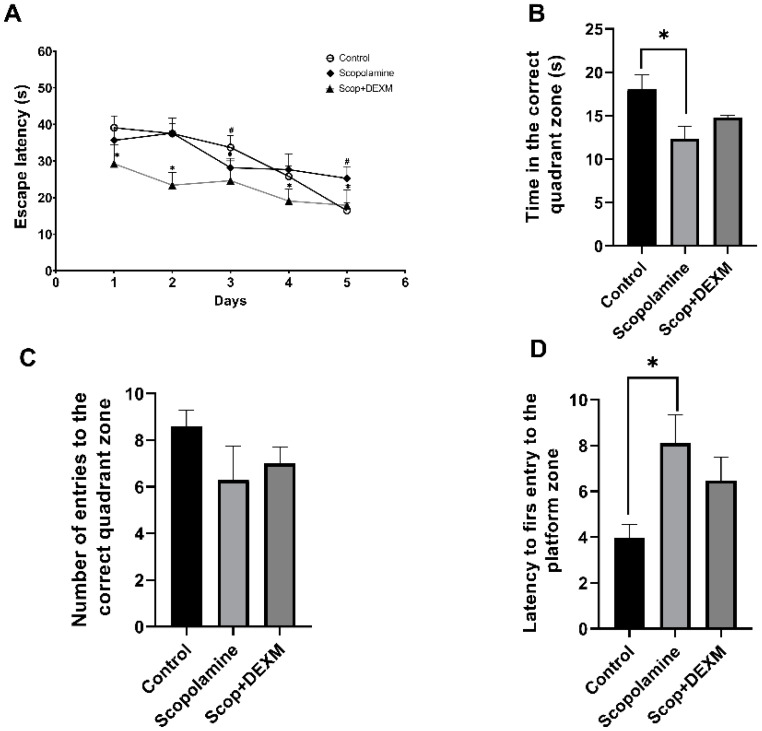
Effects of DEX on spatial memory in rats with SCOP-induced cognitive impairment. (**A**) Time spent finding the platform during the training phases. * *p* < 0.05 DEX + SCOP versus SCOP. # *p* < 0.05 SCOP versus Control. (**B**) Time spent in the target quadrant in the probe trial. (**C**) Number of entries into the quadrant with the platform. (**D**) Latency of the rats’ first entry into the quadrant with the platform. Data are presented as mean ± SEM (n = 7). Abbreviations: Dexmedetomidine (DEXM) * *p* < 0.05.

The effects of DEX on spatial memory were evaluated by the MWM test. During the acquisition phase, as shown in Figure 1, there was no statistical significance in the escape latency of SCOP-treated rats compared to the control rats on days 1, 2 and 4 (Figure 1, *p* > 0.05). However, on days 3 and 5, SCOP-treated rats had a longer escape latency compared to the control rats (*p* < 0.05). On the other hand, DEX treatment significantly reduced the rats’ escape latency from day 1 to day 5 compared to the SCOP group (Figure 1A, *p* < 0.05). In the probe phase, the time spent in the target quadrant was significantly lower in the SCOP group compared to the control group (Figure 1B, *p* < 0.05). However, the DEX treatment did not significantly change the time spent in the target quadrant (*p* > 0.05). Similarly, the number of rats entering the platform was significantly lower in the SCOP group. However, the DEX treatment did not significantly increase the number of entries into the platform region (Figure 1C, *p* > 0.05). The latency time at first entry to the platform zone was longer in the SCOP group (Figure 1D, *p* < 0.05). However, DEX administration did not shorten the latency at first entry into the target quadrant in the SCOP group (*p* > 0.05).

### 3.2. Biochemical Results

#### BDNF, ACh and AChE Analysis Results

**Figure 2:**
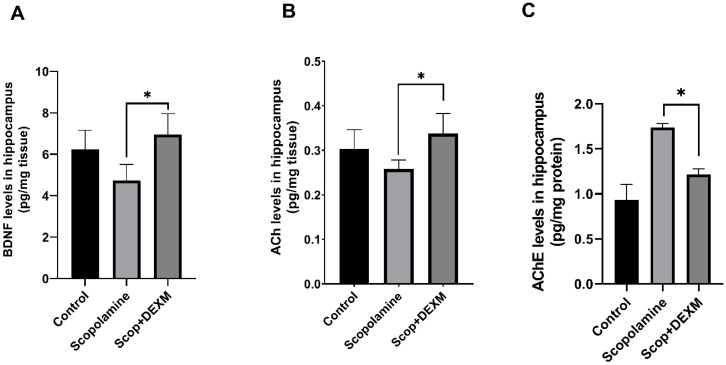
Effect of DEX on hippocampal BDNF, ACh and AChE levels in rats with SCOP-induced cognitive impairment. Following behavioral tests, the (**A**) BDNF, (**B**) ACh and (**C**) AChE levels in the hippocampus were analyzed using ELISA kits. Data are presented as mean ± SEM (n = 7). Abbreviations: BDNF, brain-derived neurotrophic factor; ACh, acetylcholine; AChE, acetylcholinesterase; Dexmedetomidine (DEXM) * *p* < 0.05.

In the present study, the BDNF, ACh and AChE levels were measured in hippocampal homogenates of rats. Accordingly, the DEX treatment significantly increased the BDNF levels compared to the SCOP group (Figure 2A, *p* < 0.05). The ACh levels were significantly increased in THE DEX-treated group compared to THE SCOP group (Figure 2B, *p* < 0.05). On the other hand, SCOP increased the AChE level, whereas the DEX treatment decreased it (Figure 2C, *p* < 0.05).

### 3.3. Histopathological Results

**Figure 3:**
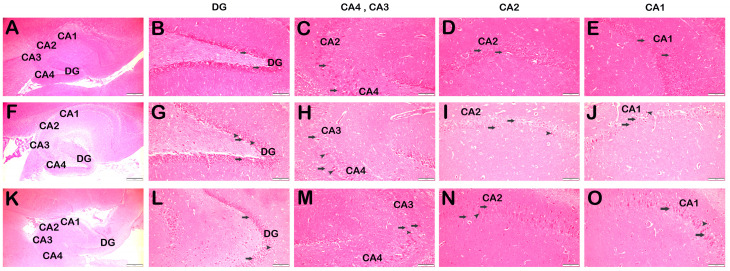
Representative light microscopic images of H+E-stained hippocampus tissue sections. (**A**) Control group: Normal neurons are observed in DG, CA1-4 structures (×4). Scale bar: 500 μm. (**B**) Control Group: DG normal neurons are observed (arrow) ×40. Scale bar: 100 μm. (**C**) Control Group: CA4 and CA3 normal neurons are observed (arrow) ×40. Scale bar: 100 μm. (**D**) Control Group: CA2 normal structure neurons are observed (arrow) ×40. Scale bar: 100 μm. (**E**) Control Group: CA1 normal neurons are observed (arrow) ×40. (HHDS: 0(0-1)). Scale bar: 100 μm. (**F**) SCOP group: DG containing ischemic neurons, CA1-4 structures are observed (×4). Scale bar: 500 μm. (**G**) SCOP Group: Many ischemic neurons are observed in the DG (arrow) ×40. Scale bar: 100 μm. (**H**) SCOP Group: CA4 and CA3 ischemic neurons are observed (arrow) ×40. Scale bar: 100 μm. (**I**) SCOP Group: Multiple ischemic neurons are observed in CA3 (arrow) ×40. Scale bar: 100 μm. (**J**) SCOP Group: Multiple ischemic neurons are observed in CA1 (arrow) ×40. (HHDS: 2(2-3)). Scale bar: 100 μm. (**K**) SCOP + DEX group: DG, ischemic neurons are decreased in CA1-4 (×4). Scale bar: 500 μm. (**L**) SCOP + DEX Group: Decreased ischemic neurons in DG (arrow) ×40. Scale bar: 100 μm. (**M**) SCOP + DEX Group: CA4 and CA3 ischemic neurons are observed to decrease in number (arrow) ×40. Scale bar: 100 μm. (**N**) SCOP + DEX Group: CA3 shows a decrease in the number of ischemic neurons (arrow) ×40. Scale bar: 100 μm. (**O**) SCOP + DEX Group: Decreased ischemic neurons are observed in CA1 (arrow) ×40. (HHDS: 1(0-1)). Scale bar: 100 μm. Abbreviations: Dentate gyrus (DT), Cornu Ammonis 1 (CA1), Cornu Ammonis 2 (CA2), Cornu Ammonis 3 (CA3), Cornu Ammonis 4 (CA4).

In the control group hippocampus sections, neurons with a typical structure were observed in DG, CA4, CA3, CA2 and CA1 (Figure 3A–E, Table 3, HHS: 0(0-0)). In contrast, the SCOP treatment group had hypertoxic necrotic neurons in DG, CA4, CA3, CA2 and CA1, with diffuse vacuoles in their cytoplasm. There were occasional edematous areas next to the necrotic neurons (Figure 3F–J, Table 3, HHS: 2(2-3)). In the SCOP + DEX treatment group, we observed a decrease in necrotic neurons and edematous areas in the DG, CA4, CA3, CA2 and CA1 parts of the hippocampus (Figure 3K–O, Table 3, HHS: 1(0-1)).

### 3.4. Immunohistochemical Results

**Figure 4:**
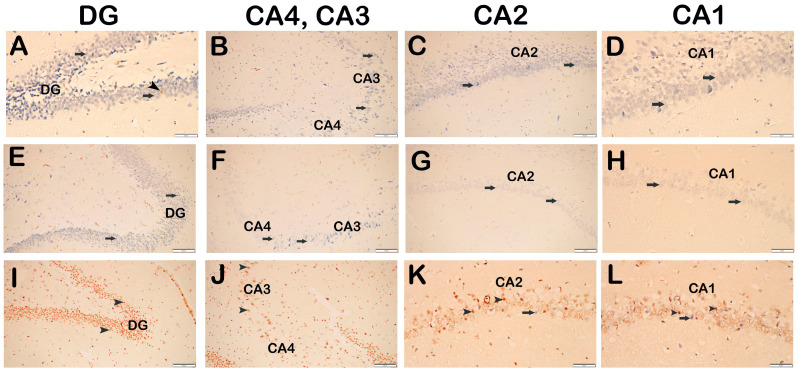
Representative light microscopic images of sections of hippocampus tissue incubated with CREB primary antibody. (**A**–**D**) Control Group: DG, CA1, CA2, CA3 and CA4 containing neurons with normal structure, slightly immunopositive for CREB primary antibody (CREB positivity score 0(0-1)). (**E**–**H**) SCOP Group: DG, CA1, CA2, CA3 and CA4 layers show a reduced number of ischemic neurons with weak CREB positivity (CREB positivity score 0(0-0)). (**I**–**L**) SCOP + DEX Group: DG, CA1, CA2, CA3 and CA4 containing a large number of neurons showing intense CREB positivity (CREB positivity score 2(1-2)). Scale bar: 50 μm (**A**,**D**,**H**,**K**,**L**), 100 μm (**B**,**C**,**E**–**G**,**I**,**J**). Abbreviations: Dentate gyrus (DT), Cornu Ammonis 1 (CA1), Cornu Ammonis 2 (CA2), Cornu Ammonis 3 (CA3), Cornu Ammonis 4 (CA4).

Sections of hippocampus tissue were labeled with CREB-positive neurons using the IHC method. In the sections of the control group, we observed that neurons with a typical structure were slightly immunopositive (Figure 4A, Table 4, CREB positivity 0(0-1)). In contrast, we observed that the neurons showing CREB positivity in the hippocampus sections of the SCOP treatment group were decreased compared to the control group (Figure 4B, Table 4, *p* = 0.001, CREB positivity 0(0-0)). In the hippocampus tissue sections of the DEX treatment group, CREB positivity cells were increased compared to the SCOP treatment group (Figure 4C, Table 4, *p* = 0.001, CREB positivity 2(1-2)).

**Figure 5:**
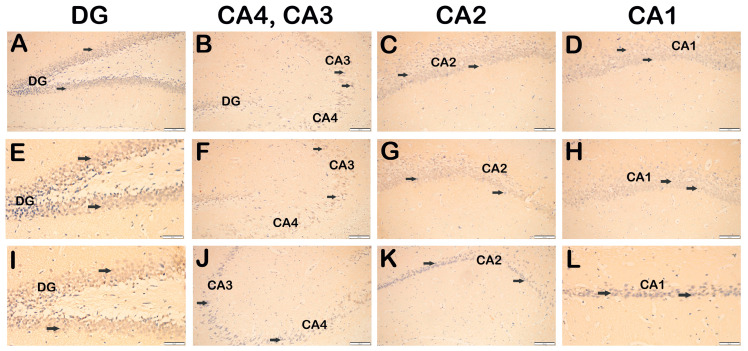
Representative light microscopic images of sections of hippocampus tissue incubated with TrkB primary antibody. (**A**–**D**) Control Group: DG, CA1, CA2, CA3 and CA4 containing normal neurons immune-negative for TrkB primary antibody (TrkB positivity score 0(0-0)). (**E**–**H**) SCOP Group: DG, CA1, CA2, CA3 and CA4 neurons immune-negative for TrkB primary antibody are observed (TrkB positivity score 0(0-0)). (**I**–**L**) SCOP + DEX Group: DG, CA1, CA2, CA3 and CA4 immune-negative neurons are observed (TrkB positivity score 0(0-0)). Scale bar: 50 μm (**E**,**I**,**L**), 100 μm (**A**–**D**,**F**–**H**,**J**,**K**). Abbreviations: Dentate gyrus (DT), Cornu Ammonis 1 (CA1), Cornu Ammonis 2 (CA2), Cornu Ammonis 3 (CA3), Cornu Ammonis 4 (CA4).

TrkB-positive neurons in the hippocampus sections were labeled using the IHC method. In the sections of the control group, we observed that the neurons with a typical structure were immune-negative (Figure 5A, Table 4, TrkB positivity 0(0-0)). Similarly, we observed that the neurons were immune-negative in the hippocampus sections of the SCOP treatment group (Figure 5B, Table 4, TrkB positivity 0(0-0)). In the hippocampus tissue sections of the DEX treatment group, we found that the neurons were immune-negative for TrkB primary antibody (Figure 5C, Table 4, TrkB positivity 0(0-0)).

## 4. Discussion

AD, the most common form of dementia, is a progressive neurodegenerative disease with a high mortality rate [24]. In addition to the existing medical treatments for the treatment of the disease, next-generation treatment protocols are also being tested. In this context, research on new drugs used for different indications in experimentally created AD models continues. In this study modelling cognitive impairment, the potential contribution of DEX, a potent α2-adrenergic receptor agonist, to the restoration of cognitive functions was investigated. The biochemical and immunohistochemical analysis results of our study indicate that DEX may play an important role in improving impaired cognitive functions.

SCOP, a muscarinic ACh receptor antagonist, easily crosses the blood–brain barrier and causes the disruption of cholinergic neurotransmission in the central nervous system. [25]. The model of cognitive dysfunction induced by SCOP is one that has been experimentally validated in numerous studies [11,22,26]. The abolition of the effect of ACh results in the loss of learning and memory functions in particular. Consistent with previous studies [1,27], in this study, it was observed that SCOP administration for 21 days caused cognitive impairment. MWM is an important way to objectively test cognitive impairment under experimental conditions in rodents [23]. The MWM test was also used in this study to determine the behavioral reflections of cognitive impairment following 21 days of SCOP administration. In the present study, a significant difference was observed between the SCOP group and the control group in terms of escape latency. These results were consistent with previous studies [28]. However, in our study, it was observed that DEX significantly changed the rats’ escape latency in the memory test. Zhang Y et al. reported that DEX improved spatial learning and memory by promoting hippocampal neurogenesis in newborn rats [29]. These different results indicate that the administration of DEX for only one week in our study was not sufficient for memory consolidation.

BDNF is an important neurotrophin that plays a key role in regulating synaptic transmission and neuroplasticity. Zhang L et al. reported that BDNF ameliorated learning deficits in a Aβ1-42-induced mouse AD model [30]. Recent studies have shown that hippocampal BDNF levels are decreased in rodents with SCOP-induced cognitive impairment [31,32]. In this study, we observed that SCOP reduced the BDNF levels in the hippocampus. On the other hand, increasing evidence suggests that BDNF may have an important role in the neuroprotective effects of DEX. In a recent study, it was reported that DEX showed beneficial effects by activating the BDNF signalling pathway in male rats with kainic acid-induced brain injury [17]. In an in vitro study, DEX-mediated neuroprotective effects in a cerebral ischemia model were shown to be accompanied by the attenuation of inflammation and oxidative stress and increased BDNF expression [19]. The present study showed that DEX increased BDNF levels. These results provide further evidence of the importance of the BDNF signaling pathway in the neuroprotective effects of DEX. In our study, we observed that the levels of TrkB, the receptor to which BDNF binds with high affinity, increased after DEX treatment. In addition, we found that the CREB levels, which mediate the transcription of genes required for neuronal survival and differentiation, also increased in our results. In a recent study, Chen et al. reported that DEX treatment may alleviate cognitive impairment in hypoxic–ischemic neonatal rats by activating hippocampal neurogenesis through the BDNF/TrkB/CREB signaling pathway [21]. Similarly, it was reported that DEX treatment reduced the attenuation of synaptic plasticity induced by sevoflurane in rats by upregulating the BDNF-TrkB-CREB signaling pathway [33]. Taken together, our study suggests that the activation of the BDNF/TrkB/CREB signaling pathway plays an important role in the treatment of SCOP-induced cognitive deficits.

ACh has a wide range of neuromodulatory effects on the cellular properties of hippocampal and cortical neurons [34]. SCOP, used in the experimental modeling of cognitive impairment, antagonizes the effects of ACh and disrupts cholinergic flow in the central nervous system, causing an AD-like pathology [35]. Previous evidence has reported a decrease in ACh levels following SCOP administration [28,36]. In addition, a recent study showed that the administration of three different doses of SCOP to rats increased hippocampal AChE activity [37]. In our study, we observed that DEX treatment increased the ACh levels in the hippocampus and decreased the AChE levels compared to the SCOP group. Although these results are consistent with our other biochemical and immunohistochemical results, some studies have reported different effects of DEX treatment on the cholinergic system. Accordingly, Nemoto et al. reported that DEX treatment did not significantly change the ACh levels in rat cortical tissues [38]. Similarly, a randomized study reported that DEX did not cause any change in AChE activity [39]. We consider the inconsistent results to be a consequence of the different tissues examined in the studies. In conclusion, we argue that additional evidence is needed to establish a direct relationship between DEX and cholinergic activity. 

This study has some limitations. Our study examined the effect of DEX on cognitive functions in the acute period. Therefore, the potential effects of chronic DEX administration should be investigated in further studies. In addition, our results should be supported by studies addressing molecular and cellular signaling pathways including the hippocampal glutaminergic system in the effects of DEX on SCOP-induced cognitive impairment.

## 5. Conclusions

This study demonstrated that DEX may play a pivotal role in the treatment of SCOP-induced cognitive dysfunction. These effects were observed to be mainly mediated by the BDNF/TrkB/CREB signaling pathway in the hippocampus.

## Figures and Tables

**Table 1:** Histopathological damage score.

Percentages	Findings	Score
**IschemicNeurons**		
**<5%**	None	0
**<25%**	Mild	1
**<50%**	Moderate	2
**<75%**	Severe	3
**Edema**		
**<5%**	None	0
**<25%**	Mild	1
**<50%**	Moderate	2
**<75%**	Severe	3

**Table 2:** Grading of the immune positivity score.

Score	
**0**	None (less than 5%)
**1**	Mild (between 6–25%)
**2**	Moderate (between 26–50%)
**3**	Severe (between 51–75%)
**4**	Very Severe (more than 76%)

**Table 3:** Histopathological Damage Scores data (median (25–75% interquartile range).

Groups	Ischemic Neuron	Edema	HHS (Median (25–75% İnterquartile Range)
**Control**	0(0-1)	0(0-0)	0(0-1)
**SCOP**	2(1.5-2) ^**a**^	0(0-1)	2(2-3) ^**a**^
**SCOP + DEX**	1(0-1) ^**a**,**b**^	0(0-0)	1(0-1) ^**b**^

^**a**^ *p* = 0.001; versus control group, ^**b**^ *p* = 0.001; versus SCOP group; Mann–Whitney U test with Bonferroni corrections.

**Table 4:** Semi-quantitative analysis score (median (25–75% interquartile range)).

Group	CREB Posivitiy Score	TrkB Positivitiy Score
Control	0(0-1)	0(0-0)
SCOP	0(0-0) ^**a**^	0(0-0)
SCOP + DEX	2(1-2) ^**b**^	0(0-0)

**^a^** *p* = 0.001; versus control group, **^b^** *p* = 0.001; versus SCOP group; Mann–Whitney U test with Bonferroni corrections.

## Data Availability

All the data generated or analyzed during this study are included in this article. The data will be available upon reasonable request (contact person: sinan.saral@erdogan.edu.tr).
